# DCDC2C Protein Expression in Human Spermatozoa: A Potential Molecular Marker for Asthenozoospermia

**DOI:** 10.3390/medsci14040449

**Published:** 2026-07-31

**Authors:** Othmane Adli, Ezzahra Rachid, Adil Kbirou, Chaymae Rochdi, Mounir Filali, Noureddine Louanjli, Said Assou, Rachid Aboutaieb

**Affiliations:** 1 Laboratory of Sexual and Reproductive Health, Faculty of Medicine and Pharmacy, Hassan II University, Casablanca 20250, Morocco; kbirou.adil@gmail.com (A.K.); raboutaieb@hotmail.fr (R.A.); 2 Science & Engineering of Biomedicals, Biophysics and Health Laboratory, Higher Institute of Health Sciences, Hassan First University of Settat, Settat 26000, Morocco; e.rachid@uhp.ac.ma; 3 Department of Urology, Ibn Rochd University Hospital Center, Casablanca 20503, Morocco; 4 Maternal-Child and Mental Health Research Laboratory, Faculty of Medicine and Pharmacy, Mohammed First University, Oujda 60000, Morocco; c.rochdi@ump.ac.ma; 5 G Lab Laboratory, Casablanca 20360, Morocco; mounirfilali@glab.ma; 6 Labomac IVF Centers and Clinical Laboratory Medicine, Casablanca 20100, Morocco; n.louanjli@gmail.com; 7 Institute for Regenerative Medicine and Biotherapy (IRMB), University of Montpellier, INSERM, CHU Montpellier, 34295 Montpellier, France; said.assou@inserm.fr

**Keywords:** asthenozoospermia, DCDC2C, sperm motility, male infertility, microtubule-associated protein, biomarker

## Abstract

**Background**: Infertility is a global public health problem among males, with asthenozoospermia representing the most common cause. Since flagellar activity is vital for proper fertilization, identification of the mechanisms controlling this process is crucial. Microtubules are necessary for sperm functionality; however, their regulatory proteins in humans require further research. DCDC2C belongs to the doublecortin domain-containing family and has a role in microtubule stabilization; therefore, DCDC2C could affect the sperm flagellar structure. **Objectives**: The goal of the present study was to examine the presence and possible relationship between DCDC2C expression and sperm motility in asthenozoospermia. **Materials and Methods**: This study involved sixty men from December 2024 to December 2025. The sperm samples were processed according to the WHO criteria (6th ed.). Conventional sperm characteristics, biochemical indicators of seminal plasma, DNA fragmentation, and chromatin condensation were evaluated. Immunofluorescence staining for DCDC2C was performed, especially with respect to flagellar localization. **Results**: DCDC2C-positive spermatozoa were significantly more common in the normozoospermic group (78.93 ± 1.67%) than in the asthenozoospermic group (17.27 ± 2.23%; *p* < 0.0001). No significant differences were found for seminal biochemical markers, sperm DNA fragmentation, or chromatin condensation. **Discussion**: Reduced detectable distal flagellar DCDC2C immunoreactivity was associated with asthenozoospermia. **Conclusions**: DCDC2C protein expression is highly correlated with sperm movement, indicating that DCDC2C is a potential molecular marker for the diagnosis of asthenozoospermia.

## 1. Introduction

Male infertility is a major health issue worldwide. It affects approximately 10–15% of couples trying to conceive, with male factors being responsible for almost half of these cases [1]. A key factor in male infertility is asthenozoospermia, which results in reduced sperm motility. Under these conditions, less than 40% of sperm cells have total motility, and under 32% show progressive motility within a semen sample [2]. This can be caused by genetic defects, environmental exposure, and lifestyle choices [3], but the exact cause is not known [4]. Since sperm motility is directly related to fertilization success, understanding the molecular mechanisms underlying asthenozoospermia would aid in good diagnostics and therapy [4,5]. Although advances have been made in reproductive medicine, many idiopathic cases of asthenozoospermia still exist; hence, the search for new molecular markers and therapeutic targets should continue [6]. An underexplored candidate related to this problem is the doublecortin domain containing 2C protein (DCDC2C). This protein has been extensively studied in neuroscience because it plays an important role in microtubule dynamics but has not been well studied in terms of male reproductive physiology. The role of DCDC2C in neuronal migration and ciliogenesis is well established; however, its potential function in human sperm with respect to flagellar integrity and motility has not been characterized [7]. Except for one paper by Fanny Jumeau and some sporadic reports, very few studies exist on the involvement of DCDC2C in human spermatozoa or its effect on motility [8]. DCDC2C is known as a regulator of microtubule polymerization and stabilization. These processes are fundamentally essential for flagellar assembly and functionality [9]. Flagellar integrity, along with the dynamic behavior of microtubules, may be a prerequisite for propulsion; thus, any aberration in DCDC2C could directly affect motility [10].

This notable information gap requires further research on the role of DCDC2C protein expression in the male reproductive system. The objective of the present investigation was therefore to analyze the differences in DCDC2C protein expression in the sperm of normal individuals and those suffering from asthenozoospermia to determine whether there is any relationship between the two. The overall aim of the project will be to estimate the DCDC2C protein expression level in the sperm of asthenozoospermic patients and normal individuals. This would help determine whether it could be used as a biomarker for both diagnosing and understanding the cause of sperm motility problems.

## 2. Materials and Methods

### 2.1. Sample Collection

The research protocol was performed in line with the Declaration of Helsinki as amended in 2013 and approved by the Institutional Ethical Committee of the University Hospital Center “20 Août” (protocol code: N° 9bis/2024; approval date: 19 February 2024).

The research was carried out with 60 men aged between 20 and 45 years who were recruited in Morocco and referred to the reproductive biology laboratories LABOMAC and GLAB and Ibn Rochd University Hospital from December 2024–December 2025. Assessment was performed on the basis of several infertility examinations and/or management via assisted reproductive technology (ART). These techniques include IUI, IVF, and ICSI.

The subjects recruited did not smoke at the time of recruitment and sampling. Where applicable, the subject had stopped smoking at least three months prior to beginning assisted reproductive technology and semen analysis.

In accordance with the guidelines provided in the World Health Organization Laboratory Manual for the Examination and Processing of Human Semen [11], all samples were obtained via masturbation following 2–5 days of sexual abstinence to guarantee preanalytical conditions. This process was carried out within the laboratory premises using sterile containers available at the laboratory.

The samples were submitted along with a clinical information sheet where relevant patient information and referral circumstances could be documented. Careful attention was given to the control of preanalytical parameters, including aseptic sample collection, proper handling of the samples, and traceability, to ensure biological integrity in the semen samples for analysis thereafter.

All semen samples were obtained following standardized protocols recommended by the WHO [11] to ensure comparability among the study population.

### 2.2. Inclusion and Exclusion Criteria

Only individuals who met certain predefined clinical and biological requirements were included in the study. The subjects were male individuals suffering from idiopathic primary infertility. Idiopathic primary infertility is characterized by a lack of identifiable causes after routine diagnostic studies have been conducted. The individuals had to be able to produce a semen sample.

To prevent protein degradation and obtain reliable molecular analyses, only samples with sperm DNA fragmentation and sperm chromatin condensation below 30% were retained. Infertility of nonobstructive origin was confirmed by seminal biochemical markers, including zinc, α-glucosidase, and fructose levels, which support normal accessory gland function.

For the asthenozoospermic group, patients were classified according to the lower reference limits reported in the WHO 6th edition manual for semen examination, with sperm motility considered reduced when progressive motility (PR) was <30% and/or total motility (PR + NP) was <42% [11].

Samples presenting with severe morphological impairment were excluded from the study. Specifically, ejaculates with a proportion of normal sperm forms less than 4%, as defined by strict morphological criteria, were not included to avoid the confounding effect of marked teratozoospermia.

Moreover, those with a clinical history of conditions that are known to affect spermatogenesis were also excluded from the present study. These criteria included individuals with a history of varicocele, azoospermia, or any other factor that could lead to male infertility.

### 2.3. Basic Sample Treatment

Immediately postharvest treatment, the semen samples were allowed to liquefy in a CO_2_ incubator at 35 °C for half an hour to provide in vivo conditions, as recommended in a laboratory setting [11]. Upon completion of the liquefication process, the sample was subjected to gentle homogenization to facilitate uniformity. The initial treatment was performed under laboratory-controlled conditions.

### 2.4. Semen Analysis

#### 2.4.1. Sperm Parameter Analysis

The physicochemical properties and functions of semen were analyzed via the latest recommendations of the OMS manual, 6th edition (July 2021) [11]. The physicochemical properties analyzed were pH, ejaculate volume (mL), sperm concentration (×106/mL), total number of spermatozoa per ejaculate (×106/ejaculate), and sperm viability percentage. The macroscopic examination included visual evaluation of the color of the semen, which should be whitish, whereas the presence of yellow coloration indicates the possible presence of infections, and a red color indicates hematospermia.

For the determination of semen pH, we used pH indicator strips 30 min post-ejaculation because the time-dependent increase in pH was avoided. The ejaculate volume was measured in a measuring tube. Motility evaluation was performed by adding 10 μL of homogenized semen to a glass slide under a light microscope (CX23 RFS1, Olympus Corporation, Tokyo, Japon) at 40× magnification.

The viability of spermatozoa was checked through eosin-nigrosin staining, allowing distinction between dead and living spermatozoa. Dead spermatozoa were stained red to pink, whereas living spermatozoa remained unstained. The sperm concentration was measured with the use of a Malassez chamber. After dilution and analysis, the following formula was applied: N = *n* × dilution factor × 1000, where N is the sperm concentration, *n* is the sperm number, and y is the number of squares.

The motility of the sperm was evaluated by mixing 10 μL of liquefied semen evenly between a slide and a cover slip. The motile pattern of the sperm was classified as progressive motile (PR), nonprogressive motile (NP), or immotile (IM) per international standards.

“Progressive motility in spermatozoa” refers to spermatozoa exhibiting progressive or large circular movement in an anterior direction, with the ability to move along the female reproductive tract. Nonprogressive motility refers to motility where the spermatozoa exhibit flagellar motion but fail to move forward, whereas motile spermatozoa lack any kind of movement.

The percentages of spermatozoa falling into each motility category were obtained from the total spermatozoa counted in several microscopic fields. Total motility was the sum of progressive and nonprogressive motile spermatozoa.

#### 2.4.2. Assessment of Sperm Morphology

Assessment of Sperm Morphology (Hematoxylin–Shorr Staining).

The morphology of the sperm cells was analyzed via the hematoxylin–Shorr stain technique, which provides clear visualization of morphological features. Smears were prepared from seminal samples on clean glass slides and dried in ambient air. The smears were fixed in 95% ethanol after air drying and stained via the hematoxylin–Shorr technique. Spermatozoa were visualized under a light microscope via immersion in oil at 100× magnification after staining. Morphological analysis was performed by examining 200 sperm cells from each seminal ejaculate, randomly chosen from the slide under a microscope.

The assessment criteria were related to major sperm structures, including the head, midpiece, and tail (flagellum). An abnormal head was defined as changes in head dimensions and the acrosomal area with specific types, such as conical, pyriform or irregularly shaped heads. The midpiece abnormalities were related to thickness and angularity, and the tail defects consisted of coiled, small, and double tails.

For each sample, the percentage of normally structured spermatozoa was estimated. The strict criterion for determining a normal sperm morphology of 4% was used following the WHO recommendations [11]; that is, normal and abnormal samples were differentiated on the basis of morphology.

This methodology allowed for precise and comparable analysis of each sample within the established criteria.

#### 2.4.3. Seminal Plasma Biochemical Analysis

The biochemical parameters of seminal plasma were assessed to evaluate the secretory function of accessory glands. After liquefaction, the samples were centrifuged (300–500× *g*, 5 min), and the supernatant (seminal plasma) was collected for analysis.

The zinc concentration was determined by using a SemenAssay^®^ Zinc Kit (BRED Life Science Technology Inc., Shenzhen, China). Measurements were based on the 5-Br-PAPS colorimetric method. Zinc is a marker for prostatic function. On the basis of reference values, the normal seminal zinc concentration has been described as ≥2.4 µmol/ejaculate [12].

Neutral α-glucosidase activity was determined via the use of the SemenAssay^®^ NAG Kit (BRED Life Science Technology Inc., Shenzhen, China). The test was based on a modified Cooper’s method. NAG is a specific marker of epididymal function; therefore, a value ≥20 mU/ejaculate is considered normal [13].

The fructose concentration was measured with the SemenAssay^®^ Fructose Kit (BRED Life Science Technology Inc., Shenzhen, China), which uses an enzymatic colorimetric method. Fructose indicates vesicular function, and normal values are defined as ≥13 μmol/ejaculate [14].

All assays were performed according to the manufacturer’s instructions with microplate-based detection. These biochemical markers help exclude patients with accessory gland dysfunction and ensure the inclusion of cases due to idiopathic infertility.

#### 2.4.4. Analysis of the Quality of the Genetic Material in Spermatozoa

##### Sperm DNA Fragmentation

There are several protocols involved in the implementation of the terminal deoxynucleotidyl transferase dUTP nick end labeling (TUNEL) method, including sample preparation steps. The sperm samples were first fixed and permeabilized by mixing with PBS and the modification solution. Centrifugation was performed, and the pellet was collected, after which it was used in preparation of the smear. Drying was then performed using a heating plate maintained at 36 °C. Afterwards, the fixation solution (37 °C formaldehyde + PBS) was added, and the slides were incubated at room temperature for 30 min. After the samples were rinsed with PBS, permeabilization solution (Triton X + citrate + distilled water) was added to the slides, which were subsequently incubated at room temperature for 1 min. Once the samples were further rinsed with PBS solution and dried, the labeling solution was added. The solution contained a TUNEL reaction mixture (In Situ Cell Death Detection Fluorescein, Roche^®^ Diagnostics GmbH, Mannheim, Germany). Coverslips were placed into the smears and incubated in the dark at 37 °C for 45 min [15]. This incubation facilitates the enzymatic addition of fluorescein-conjugated deoxyuridine triphosphate to the 3′-hydroxyl ends of fragmented DNA by terminal deoxynucleotidyl transferase [3,16]. We used DNA fragmentation caused by DNase I as a positive control and a negative control, where the TdT enzyme was left out of the labeling solution to check for background fluorescence [15,17]. After rinsing with a modified PBS solution, drops of glycerol were placed between the slides and coverslips (all steps were performed in the dark). Fluorescence microscopy was then used to visualize and quantify DNA fragmentation on the slides. This method allows the visualization of sperm cells with fragmented DNA and positive fluorescence [18]. To determine the percentage of sperm cells that possess broken DNA, the number of stained cells is divided by the total number of cells [19,20]. The quantitative measurement of the percentage of TUNEL-positive cells is a crucial indicator of sperm DNA quality [21].

##### Sperm Chromatin Condensation

The procedure outlined above provides guidelines on how to conduct the assay of defects in sperm chromatin packaging via aniline blue staining, improving existing procedures for the assessment of sperm DNA fragmentation. Aniline blue staining selectively stains lysine-rich histones and does not stain arginine/cysteine-rich protamines; therefore, it is possible to detect spermatozoa with compromised chromatin structures [3]. The traditional assay involves fixation and permeabilization of the sperm and aniline blue staining, followed by rinsing the slides and drying [3]. This staining allows for the identification of spermatozoa with immature or poorly condensed chromatin, characterized by abnormal histone retention and increased susceptibility to aniline blue [22]. To determine the percentage of spermatozoa with decondensed chromatin, it is necessary to count the number of stained cells and compare them to the total number of cells present [23]. Abnormalities in chromatin condensation may be considered when the majority (over 50%) of the heads of the spermatozoa exhibit dark blue staining. Properly protaminated sperm cells exhibit light blue or faint pink staining [24].

#### 2.4.5. Immunofluorescence Analysis

The sperm slides were prepared with 5 to 10 µL of purified sperm suspension applied to the slides. The drying process was performed under an air atmosphere for 10 to 15 min, and the samples were further subjected to mild heat fixation to enhance the adherence of the spermatozoa. The purified spermatozoa were washed twice with 1× PBS after being centrifuged at 300 to 500× *g* for 5 min to eliminate residual seminal plasma.

The slides were subjected to cold acetone fixation (−20 °C) for 5 min, followed by two washes with 1 × PBS for 5 min each. Blocking of nonspecific binding sites was achieved by incubating the slides in PBS containing 1% bovine serum albumin (BSA) for 30–45 min at room temperature in a humid chamber [8].

The slides were incubated overnight in PBS containing 1% BSA together with a rabbit polyclonal anti-DCDC2C antibody (ab126564, Abcam, Cambridge, UK) diluted 1:200. Antibody specificity was previously validated by Jumeau et al. (2017), who demonstrated flagellar DCDC2C expression in human spermatozoa via immunofluorescence with this antibody [8].

The slides were rinsed three times in PBS (5–10 min each), followed by incubation for 90 min in the dark with Alexa Fluor 488-conjugated goat anti-rabbit IgG (Invitrogen, Waltham, MA, USA) diluted 1:600 in PBS.

Finally, the slides were mounted with glycerol-based mounting medium (PBS: glycerol, 1:1) and observed under a fluorescence microscope. Negative controls were generated by omitting the primary antibody.

Fluorescence analysis was performed on a fluorescence microscope equipped with a FITC filter set (excitation ~488 nm; emission ~520 nm). Images were taken at ×1000 magnification via oil immersion. For quantitative analysis, at least 200 spermatozoa per sample were evaluated, and the percentage of DCDC2C-positive spermatozoa was calculated on the basis of the presence of a fluorescent signal at the distal extremity of the flagellum.

The anti-DCDC2C antibody (ab126564, Abcam) was previously validated by the manufacturer for immunofluorescence applications. Specificity was confirmed via the use of negative controls in which the primary antibody was omitted.

At least 200 spermatozoa per sample were analyzed independently by two experienced technicians, both of whom were blind to the assignment of the clinical groups at the time of image analysis.

### 2.5. Statistical Analysis

Statistical analyses were performed via GraphPad Prism 10 software. The data are presented as the means ± SEMs. The normality of the data distribution was assessed via normality tests and QQ plot inspection. As the data followed an approximately normal distribution, differences between groups were analyzed via an unpaired two-tailed Student’s *t* test. A *p* value < 0.05 was considered statistically significant.

A post hoc power analysis was performed on the basis of the observed effect size for DCDC2C expression (Cohen’s d = 5.72), with α = 0.05 and power = 0.80, indicating that the sample size (*n* = 30 per group) was sufficient to detect between-group differences.

Given the exploratory nature of the study, the limited sample size, and the highly selected cohort, the statistical analysis was limited to univariate comparisons.

## 3. Results

### 3.1. Seminal Plasma Biochemical Analysis

**Figure 1:**
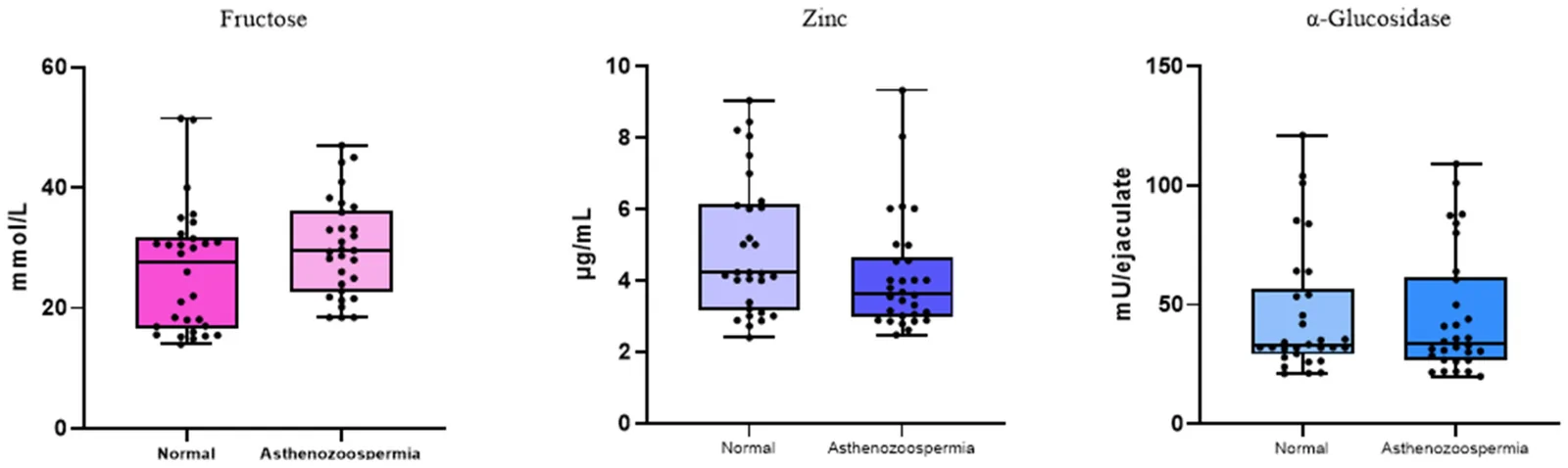
Seminal plasma biochemical profile in normozoospermic and asthenozoospermic groups. Analysis of seminal biochemical markers revealed no significant differences between normozoospermic and asthenozoospermic patients. The levels of fructose, zinc and α-glucosidase were comparable between the two groups. Statistical analysis via unpaired two-tailed Student’s *t* tests confirmed the absence of significant differences in these parameters (*p* = 0.6164, *p* = 0.9351, *p* = 0.6637, respectively) (Appendix A.1).

Seminal plasma biochemical markers, fructose, zinc, and α-glucosidase, were assessed to evaluate accessory gland function in normozoospermic and asthenozoospermic patients. Seminal fructose concentrations were comparable between the two groups, with no statistically significant difference (*p* = 0.6164). Likewise, zinc levels, did not differ significantly between normozoospermic and asthenozoospermic patients (*p* = 0.9351). α-Glucosidase activity, was also similar between groups (*p* = 0.6637). Taken together, these results suggest that the biochemical secretory function is preserved in asthenozoospermic patients and does not appear to be directly associated with impaired sperm motility (Figure 1). 

### 3.2. Analysis of the Quality of the Genetic Material in Spermatozoa

#### 3.2.1. Sperm DNA Fragmentation

**Figure 2:**
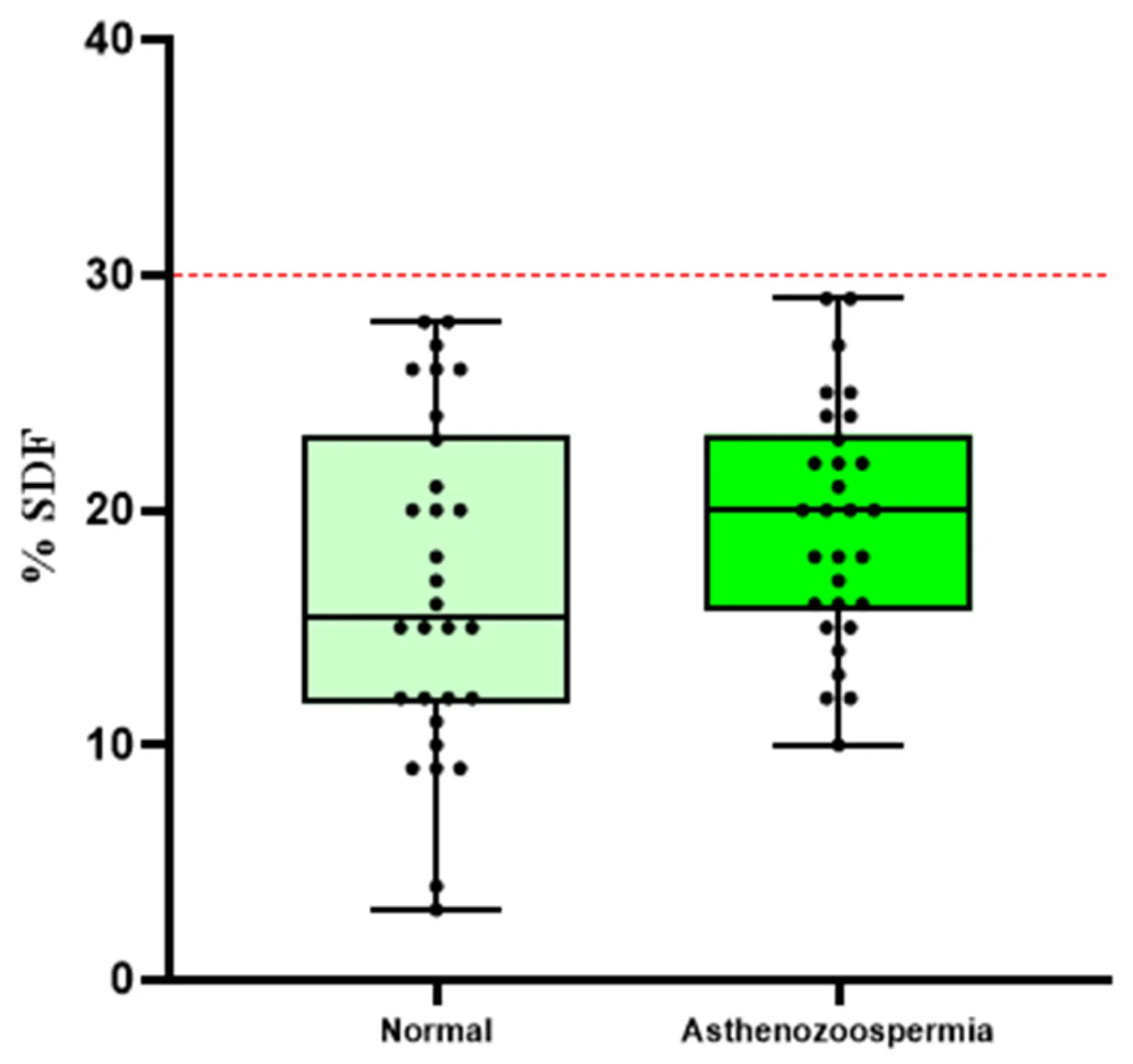
Sperm DNA fragmentation in normozoospermic and asthenozoospermic groups. Analysis of sperm DNA fragmentation (SDF) revealed no significant difference between normozoospermia and asthenozoospermia patients. The percentage of SDF was comparable between the two groups, with mean values of 16.77% in the normozoospermia group and 19.43% in the asthenozoospermia group. Statistical analysis via an unpaired two-tailed Student’s *t* test confirmed the absence of a significant difference between groups (*p* = 0.0996). In both groups, the SDF values remained below the clinical threshold of 30%. The red line represents the normal threshold, which is equal to 30%.

The percentage of sperm DNA fragmentation (SDF) was comparable between normozoospermic and asthenozoospermic patients, with mean values of 16.77% and 19.43%, respectively, and no significant difference (*p* = 0.0996) (Figure 2). Notably, SDF values in both groups remained below the 30% clinical threshold associated with an increased risk of adverse reproductive outcomes, indicating that the genomic integrity of spermatozoa is not markedly compromised in asthenozoospermic patients despite their reduced motility.

#### 3.2.2. Sperm Chromatin Condensation

**Figure 3:**
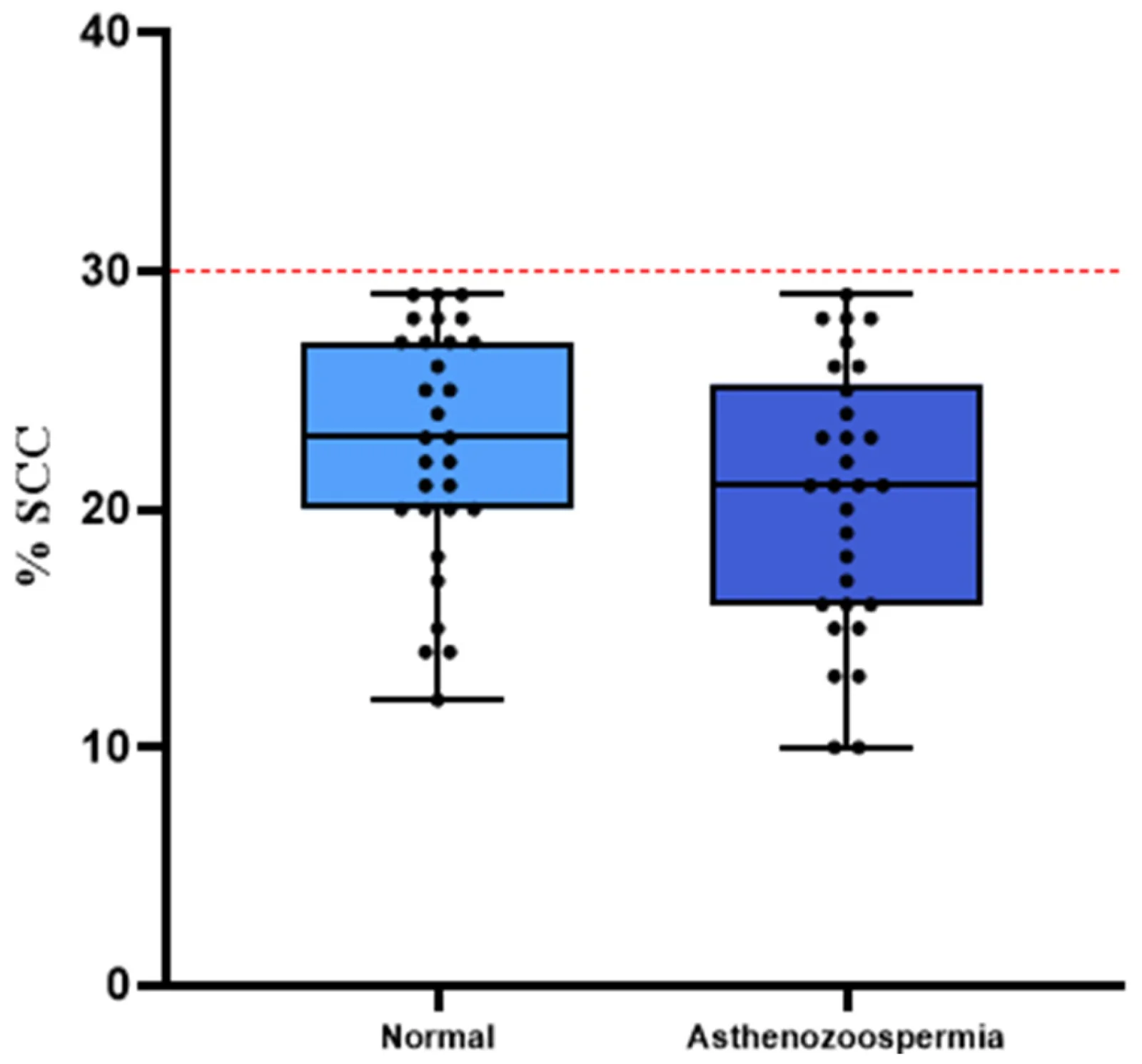
Sperm chromatin condensation in normozoospermic and asthenozoospermic groups. According to a study on sperm chromatin condensation, there was no statistically significant difference between men with normozoospermia and those with asthenozoospermia. The percentage of SCC was almost equal between the two groups of individuals, with 22.70% for normozoospermic men and 20.47% for asthenozoospermic men. According to an unpaired two-tailed Student’s *t* test, there was no statistically significant difference between the two groups (*p* = 0.0871). The red line represents the normal threshold, which is equal to 30%.

The percentage of sperm chromatin condensation (SCC) was almost equal between normozoospermic and asthenozoospermic men, at 22.70% and 20.47%, respectively, and no significant difference (*p* = 0.0871) (Figure 3). In both groups, SCC values remained below the 30% clinical threshold, indicating preserved chromatin condensation in spermatozoa from asthenozoospermic patients.

### 3.3. Immunofluorescence Analysis

**Figure 4:**
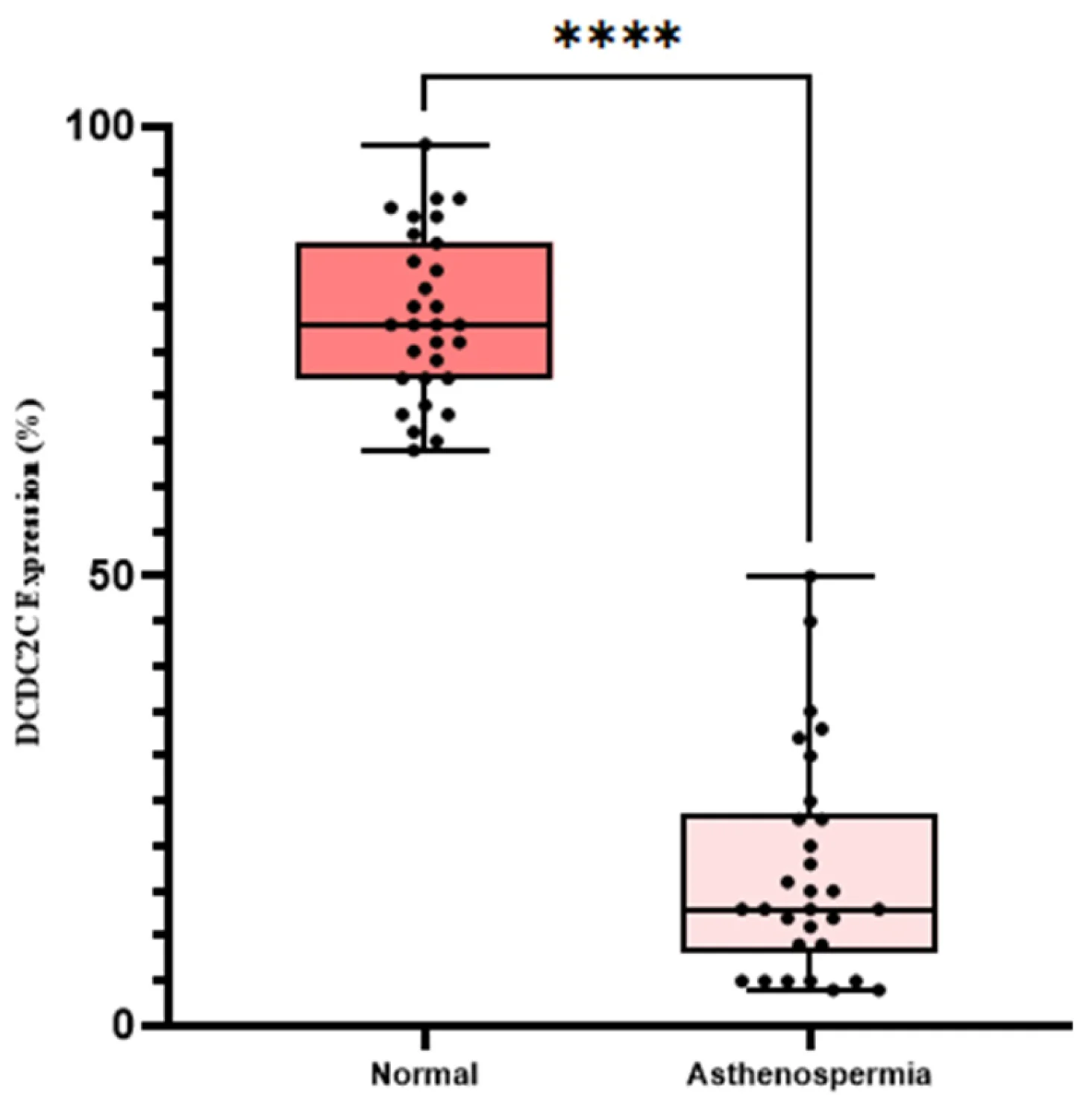
Reduced distal flagellar DCDC2C immunoreactivity in spermatozoa from asthenozoospermic men. Immunofluorescence analysis revealed a marked difference in the proportion of DCDC2C-positive spermatozoa between normozoospermic and asthenospermic patients. Quantitative assessment revealed that the percentage of DCDC2C-positive spermatozoa was significantly greater in the normozoospermic group than in the asthenospermic group (78.93 ± 1.67% vs. 17.27 ± 2.23%; *n* = 30 per group). Statistical analysis via an unpaired two-tailed Student’s *t* test revealed a significant difference between the two groups (**** *p* < 0.0001). Notably, the distribution of individual values clearly differed between groups, with consistently high DCDC2C expression observed in normozoospermic samples and markedly reduced expression in asthenospermic samples (Appendix A.2).

**Figure 5:**
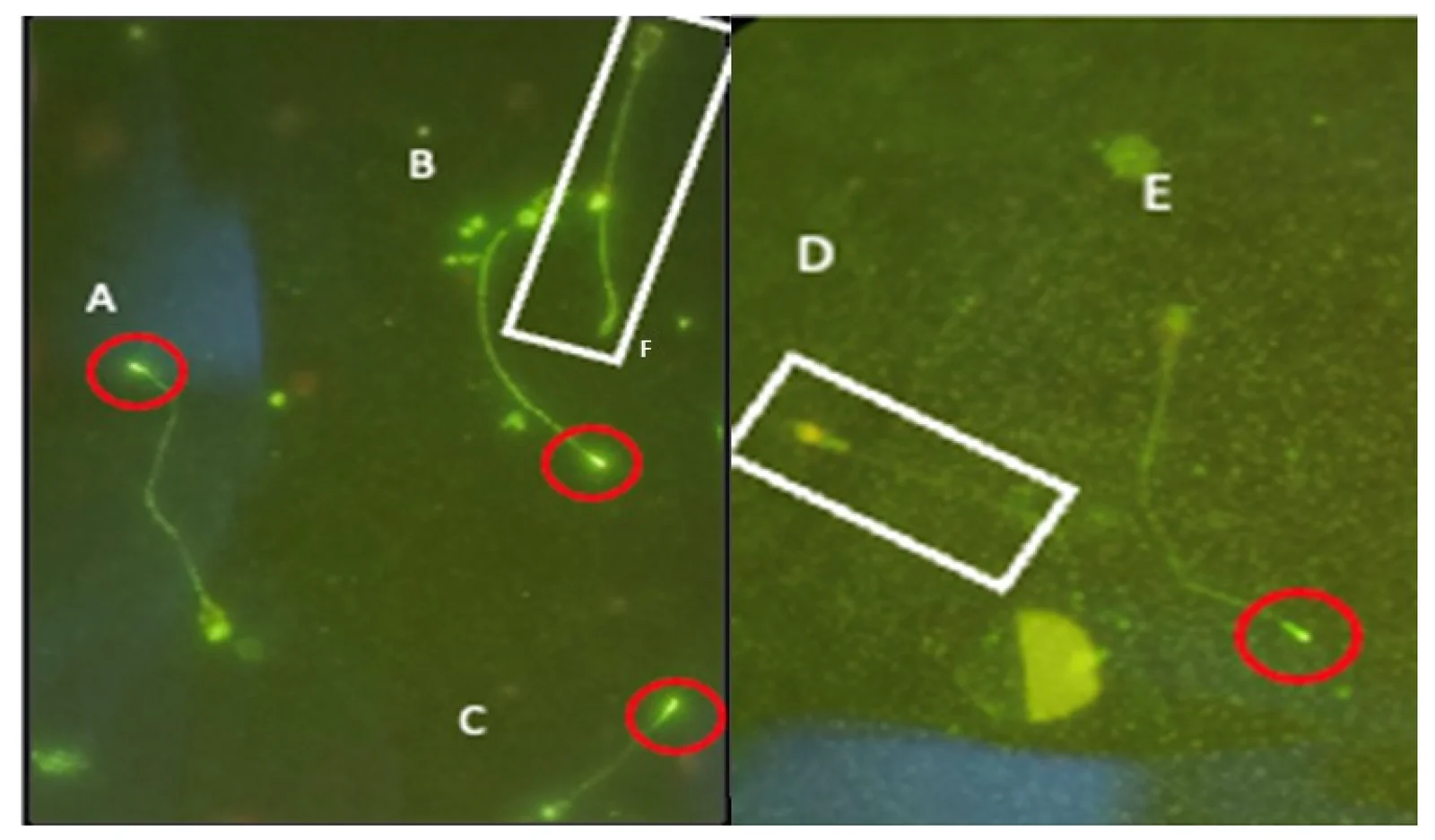
Distal flagellar localization of DCDC2C in human spermatozoa. Immunofluorescence using anti-DCDC2C antibodies revealed a unique staining pattern at the distal end of the sperm flagellum. As seen in A–C and E, a unique fluorescence is observed at the distal tip of the flagellum (circled in red), indicating DCDC2C presence. D and F where a spermatozoon is circled in white, there is no unique fluorescence detected in the distal end of the flagellum, indicating DCDC2C absence. It is evident that DCDC2C exhibits a heterogeneous expression pattern within spermatozoa and prefers distal flagellar localization when expressed.

The percentage of DCDC2C-positive spermatozoa was significantly greater in the normozoospermic group than in the asthenospermic group (78.93 ± 1.67% vs. 17.27 ± 2.23%; *n* = 30 per group; *p* < 0.0001) (Figure 4). Immunofluorescence localization further showed that DCDC2C labeling was consistently confined to the distal end of the flagellum when present, whereas spermatozoa lacking this signal showed no detectable distal flagellar fluorescence (Figure 5). The clear separation observed between the two groups suggests that DCDC2C expression could represent a promising indicator of asthenospermia.

The clear separation observed between the two groups suggests that DCDC2C expression could represent a promising indicator of asthenospermia.

## 4. Discussion

The purpose of the present study was to elucidate the relationship between DCDC2C expression and asthenozoospermia. Specifically, in this study, 60 semen samples were obtained from male patients who suffered from idiopathic primary infertility. The purpose of this work was to evaluate their characteristics according to sperm DNA fragmentation (SDF), sperm chromatin condensation (SCC), semen biochemical analysis, sperm characteristics, and morphology, as well as DCDC2C protein expression levels. The above tests were carried out via the TUNEL test, aniline blue stain test, 5-Br-PAPS colorimetry, modified Cooper test, enzymatic colorimetry, spermogram, spermocytogram, and immunofluorescence.

The study outcomes revealed significant discrepancies, highlighting, to our knowledge, a strong association between routine sperm parameters and a decrease in the expression level of the structural protein DCDC2C. Our findings thus show that a reduction in DCDC2C could be a potential marker of idiopathic infertility [8,25]. At the same time, our findings prove once again that, despite the significance of secondary testing, such as seminal biochemistry, sperm DNA fragmentation (SDF), and sperm chromatin condensation (SCC), the latter are still far from exhaustive investigations, especially in the case of unexplained asthenospermia [26].

### 4.1. Seminal Biochemistry

The results of the statistical analyses of the three hallmark biochemical markers associated with seminal vesicle function (Zn^2+^), prostatic function (fructose), and epididymal function (α-glucosidase) were not significantly different between the asthenospermic groups, with *p* values of 0.6164, 0.9351, and 0.6637, respectively. This finding is biologically sound because changes in seminal plasma biochemistry have been traditionally associated with cases of infertility with an obstructive etiology, because of the involvement of accessory gland dysfunction or duct obstruction [27,28,29]. Because there was no obstructive pathology in any of the patients included in this study, it is reasonable to assume that these biochemical markers were intact. In addition, the lack of a significant correlation between these biochemical markers and DCDC2C protein expression implies that the lower levels of DCDC2C protein expression are due to another mechanism, different from secretory action.

### 4.2. SDF and SCC

Statistical analysis revealed no significant differences in SDF or SCC between patients with lower DCDC2C protein expression and those with normal DCDC2C protein expression (*p* = 0.0996 and *p* = 0.0871, respectively). This result was expected, as subjects with SDF or SCC levels exceeding 30% were excluded from the study. Elevated SDF and abnormal SCC are recognized as indirect markers of oxidative stress [3], which is known to nonselectively damage structural proteins through oxidative degradation. When these subjects are included, their SDF and SCC values act as confounders due to the influence of oxidative stress on DCDC2C expression [26], making it impossible to determine whether reduced DCDC2C expression is directly associated with asthenozoospermia or is instead mediated by oxidative stress.

In the current study population, the link between reduced DCDC2C signal and asthenozoospermia occurred in the absence of increased sperm DNA damage and chromatin abnormalities. Nonetheless, due to the fact that these factors have been controlled in the experiment, more studies will be needed to confirm this correlation.

### 4.3. DCDC2C and Asthenospermia

Semiquantitative analysis revealed highly significant differences in DCDC2C protein expression between the asthenospermic cohort and the normospermic control cohort (*p* < 0.0001), thus indicating the direct connection between sperm motility and DCDC2C protein expression levels. This result is biologically plausible, considering the established function of the DCDC (doublecortin domain-containing) protein family in maintaining the proper functioning of specialized membrane-bound organelles found on the surface of certain cells capable of generating movement, owing to the unique axonemal structure [8]. The structure consists of a microtubular assembly organized in the characteristic 9 + 2 pattern, whereby nine peripheral microtubules surround the central pair [30]. Similarly, the axoneme structure is crucial for the proper functioning of the sperm flagellum, which is responsible for sperm motility [31].

Notably, DCDC2C expression was detected in less than 50% (range 4–50%) of all the samples with asthenospermia (30 samples), whereas DCDC2C expression was detected in more than 64–98% (all 30 control samples). In this way, there is no overlap between the two groups, which can be considered strong evidence of a threshold effect, meaning that DCDC2C expression is needed to maintain proper axoneme structure and, consequently, sperm motility [25]. Additionally, the total absence of overlap between the two groups may increase the usefulness of DCDC2C as a marker for diagnosing idiopathic asthenospermia.

Despite its biological importance, very little research has been done on this subject in the existing literature [32]. To date, only a small amount of research has been conducted on this relationship, such as studies that have shown that circRNAs from DCDC2C are directly related to the motility of sperm in pigs [33]. While animal studies provide an interesting scientific basis for understanding this phenomenon, very little research has been conducted on this relationship in humans [34].

## 5. Limitations of the Study

This descriptive study demonstrates a relationship between decreased DCDC2C expression and asthenozoospermia; however, the causative relationship remains unproven. Further functional studies will be necessary to clarify any relationship between DCDC2C and the functioning of the flagellum.

A limitation of the present study is the selectivity of the study sample. The asthenozoospermic group consisted of those men suffering from isolated asthenozoospermia as opposed to the whole asthenozoospermic population seen in daily medical practice. This selective sampling was deliberately chosen since the aim was to minimize the presence of confounders and explore the association between DCDC2C expression and sperm motility in cases of idiopathic asthenozoospermia. Consequently, the findings cannot be generalized. It implies that the current findings cannot be applied to cases of asthenozoospermic patients who have varicocele, severe teratozoospermia, high sperm DNA fragmentation, chromatin abnormalities, abnormal biochemical parameters of the semen, or other known causes of male infertility.

## 6. Conclusions

The results of the present study revealed an association between decreased DCDC2C protein expression and idiopathic asthenospermia, suggesting that DCDC2C may represent a potential new biomarker for the diagnosis of male infertility. The total exclusion of any DCDC2C expression overlap between asthenospermic and normospermic populations shows that the former DCDC2C expression is lower than 50%, whereas the latter, whose values were consistently above 64%, indicates the presence of a definite threshold of expression essential for sustaining flagellar axonemal structure and functionality. Most importantly, the lack of differences in seminal biochemical profiles and oxidative stress indicators demonstrates that decreased DCDC2C expression occurs through a completely novel molecular mechanism unrelated to secretory or oxidative processes. Therefore, the findings indicate that current standard second-line examinations can provide only limited data and should be complemented by examination of DCDC2C expression.

The results of this study open several promising avenues for future research. First, the validation of DCDC2C as a clinical marker should be conducted with larger samples of patients in multicenter trials, especially with the aim of identifying a specific expression threshold, which was not the purpose of the current explorative study. The second direction of studies should concern the creation of standardized protocols for measuring DCDC2C expression in sperm on the basis of immunofluorescence. This will make it possible to include it among the markers routinely used during andrological tests. Third, investigating the mechanism through which the DCDC2C gene affects axonemal integrity via proteomic and ultrastructural methods is necessary. Finally, the hypothesis that low expression of this protein in sperm indicates the genetic vulnerability of men to dyslexia should be tested in dedicated studies, with special attention given to identifying the relationship between the father’s sperm DCDC2C expression and germline variants of this gene in dyslexic children. Confirmation of this assumption would dramatically change the approach to genetic counseling and could make the standard fertility test an effective screening method.

## 7. Perspective

In addition to its involvement in sperm motility discussed above, DCDC2C is a member of the doublecortin domain-containing (DCDC) family of proteins, characterized by their common domains responsible for microtubule binding and cytoskeletal organization, as well as for ciliary and flagellar function [35,36]. Specifically, one DCDC family member, namely DCDC2, located within the DYX2 locus of chromosome 6p22, has been linked to the presence of developmental dyslexia and related phenotypes in multiple populations independently [35,37]. The linkage between the gene and the condition is explained partially by the involvement of the doublecortin domain in microtubule dynamics and ciliary function in neurons [36,38,39]. However, the current study did not address genetic variants of DCDC2 and DCDC2C genes, inheritance of DCDC2C expression from the father to his offspring, and neurodevelopmental outcomes of interest, and there is no information available to suggest the existence of any connection between decreased or absent sperm DCDC2C expression and dyslexia risk in children of such men. Nevertheless, it is possible that the DCDC2C expression observed in the spermatozoa may be indicative of some broader changes related to microtubules and cilia functioning [40,41].

## Data Availability

The original contributions presented in this study are included in the article Further inquiries can be directed to the corresponding author.

## Abbreviations

The following abbreviations are used in this manuscript:

ART	Assisted Reproductive Technology
AURKC	Aurora Kinase C
CASA	computer-assisted sperm analysis system
FTs	typical forms
ICSI	intracytoplasmic sperm injection
IUI	intrauterine insemination
IVF	in vitro fertilization
ROS	Reactive oxygen species
SCC	Sperm Chromatin Condensation
SDF	Sperm DNA Fragmentation
TZI	teratozoospermia index

## Appendix A

### Appendix A.1

**Table A1:** Seminal biochemical parameters in the normozoospermic and asthenozoospermic groups (means ± SEMss).

Group	*n*	Fructose	Zinc	α-Glucosidase
**Normal**	30	26.27 ± 1.90	4.922 ± 0.35	45.99 ± 4.96
**Asthenozoospermia**	30	29.99 ± 1.48	4.13 ± 0.29	45.42 ± 4.76
***p* value**		0.6164	0.9351	0.6637

### Appendix A.2

**Table A2:** Quantitative analysis of DCDC2C-positive spermatozoa in normozoospermic and asthenozoospermic groups.

Group	*n*	Min	Max	Mean ± SEM (%)	*p* Value
**Normal**	30	64	98	78.93 ± 1.67	****
**Asthenozoospermia**	30	4	50	17.27 ± 2.23	

**** *p* < 0.0001 asthenozoospermia vs. normal (unpaired two-tailed Student’s *t* test).
